# Treatment of Aneurism by Compression

**Published:** 1847-04

**Authors:** 


*Treatment of Aneurism by Compression.*—Dr. Bellingham gives a decided preference to the treatment of aneurism by compression, for the following reasons: 1. That the mode in which consolidation of the aneurism is brought about by compression is exactly the same as that in which a natural or spontaneous cure occurs. 2. Because when a cure is effected by compression, the vessel is obliterated merely at the site of the aneurism; whereas when a ligature is applied in the usual situation, at some distance from the tumor, the artery is obliterated both at the seat of the ligature and at the seat of the aneurism. He looks upon the treatment by compression as safer than that of ligature.—*Ibid.—Medical News.*

